# Recommendations Favoring Anal Cytology as a Method for Anal Cancer Screening: A Systematic Review

**DOI:** 10.3390/cancers11121942

**Published:** 2019-12-04

**Authors:** Andreia Albuquerque, Elisabete Rios, Fernando Schmitt

**Affiliations:** 1 Faculty of Medicine of the University of Porto, 4200-319 Porto, Portugal; culex.rios@gmail.com (E.R.); fschmitt@ipatimup.pt (F.S.); 2 Gastroenterology Department St. James’s University Hospital, Leeds LS9 7TF, UK; 3 Pathology Department Centro Hospitalar São João, 4200-319 Porto, Portugal; 4 Institute of Molecular Pathology and Immunology of the University of Porto (IPATIMUP), 4200-135 Porto, Portugal

**Keywords:** anal cytology, anal cancer, screening

## Abstract

Clinicians are increasingly facing the decision of performing anal cancer screening in high-risk groups. Anal cytology is commonly the first approach. We systematically reviewed recommendations favoring anal cytology for anal cancer screening. Three databases were searched: PubMed, Scopus, and Embase, from January 2007 to 12 September 2019. The references cited by the retrieved articles and the websites of relevant organizations were also searched without language restrictions. Studies reporting guidelines from regional or national societies, institutes, or groups were included. Eight papers met the inclusion criteria and were selected, five were from the United States of America (USA) and three from Europe. There were no national recommendations published. There was one guideline specifically for solid-organ transplant recipients. The other seven targeted HIV-positive patients, with HIV-positive men who have sex with men (MSM) included as a screening group in all of these. Two recommendations favored screening in all HIV-positive patients. Five recommendations targeting HIV-positive patients made considerations about the cytology follow-up, recommending at least annual cytology in case of a normal result, and in case of squamous cytological abnormalities, a referral for anoscopy/high-resolution anoscopy. There were no recommendations for upper and lower age limits for screening. In conclusion, several societies recommend anal cancer screening using anal cytology in HIV-positive MSM patients. There is a lack of screening recommendations for other high-risk groups, with only one society recommending screening in transplant recipients.

## 1. Introduction

Anal squamous cell carcinoma (SCC) is a malignancy associated with anal human papillomavirus (HPV) infection [1], the incidence and mortality of which have been and will continue to increase [2,3]. The highest-risk group is HIV-positive men who have sex with men (MSM). According to a meta-analysis by Machalek et al. [4], including nine studies from both the era before highly active antiretroviral therapy (HAART) and the HAART era, the incidence rate (IR) in HIV-positive MSM was 45.9 per 100,000 men. The incidence of anal SCC was higher in the HAART era, 77.8 per 100,000 men vs. 21.8 per 100,000 men pre-HAART [4], given the improved survival results and therefore a longer possible exposure to HPV infection [5]. Anal SCC is one of the most common non-AIDS-defining cancers [6] and the most common HPV-driven cancer [7], in high-income settings, in HIV-positive patients. Less information related to anal SCC incidence has been published for other groups, but data showed an absolute IR of 12.3 per 100,000 person-years in solid-organ transplant recipients [8]. In HIV-negative MSM, the IR was 5.1 per 100,000 men [4], and the described annual incidence of anal cancer in the general population was 1.9 per 100,000 person-years [9].

Anal and cervical cancers have the same etiological agent (HPV) and the same type of squamous intraepithelial precancerous lesions, cervical intraepithelial neoplasia (CIN) and anal intraepithelial neoplasia (AIN), respectively. There is a known sequence from persistent HPV infection to low- to high-grade squamous intraepithelial lesions (HSIL), and finally, to invasive cancer [10]. A screening process similar to the one for the cervix has been described for the anus, using anal cytology as the first screening approach, with referral of those with abnormalities to high-resolution anoscopy (HRA).

There is still a lack of consensus recommendations for anal cancer screening in those at highest risk. Health care professionals providing anal cancer screening most often use anal cytology [11] and require an abnormal anal cytology prior to performing HRA in asymptomatic patients [12]. The sensitivities of anal and cervical cytology are comparable [13,14], and the sensitivity of anal cytology for the detection of anal HSIL in immunosuppressed populations (who could benefit more from screening) has been shown to be high [14].

Given the increasing incidence and mortality of anal SCC [2,3], in the future, clinicians will likely face a greater need to decide whether and how to conduct screening in the identified high-risk groups (e.g., HIV-positive MSM, transplant recipients), who are experiencing increasing survival rates and therefore greater exposure to HPV complications [5,15]. An understanding of the current guidelines supporting anal cancer screening can be relevant for clinicians. We systematically reviewed national and regional guidelines to evaluate recommendations favoring anal cytology for anal cancer screening.

## 2. Methods

The study was performed according to the Preferred Reporting Items for Systematic Reviews and Meta-analyses (PRISMA) recommendations [16]. 

Two authors (A.A. and E.R.) searched three electronic databases (PubMed, Scopus, and Embase) for articles published between January 2007 and 12 September 2019. We used the terms “anus” AND “neoplasms” AND “guidelines”. The references cited by the retrieved articles were also evaluated to identify other relevant studies. We also searched the websites of relevant professional organizations. In cases of discrepancy, a consensus was reached, and no disagreements required adjudication. There was no language restriction. Only studies reporting recommendations in favor of anal cytology for anal cancer screening in adult patients, from regional and national societies, institutes, and groups, were included. Recommendations for using anal cytology in the detection of anal dysplasia after anal cancer diagnosis were not included. Guidelines that stated that some specialists do screen high-risk patients for anal dysplasia using anal cytology, but that did not make a formal recommendation for the use of anal cytology were not included. Recommendations advocating for anal cancer screening, but not including anal cytology as a method of screening, were not included.

Information on the society/institute/group, year, country, high-risk group, type of recommendation, screening age, management after cytology, grade of the recommendation, and HPV testing was collected and presented in Table 1.

## 3. Results

**Figure 1:**
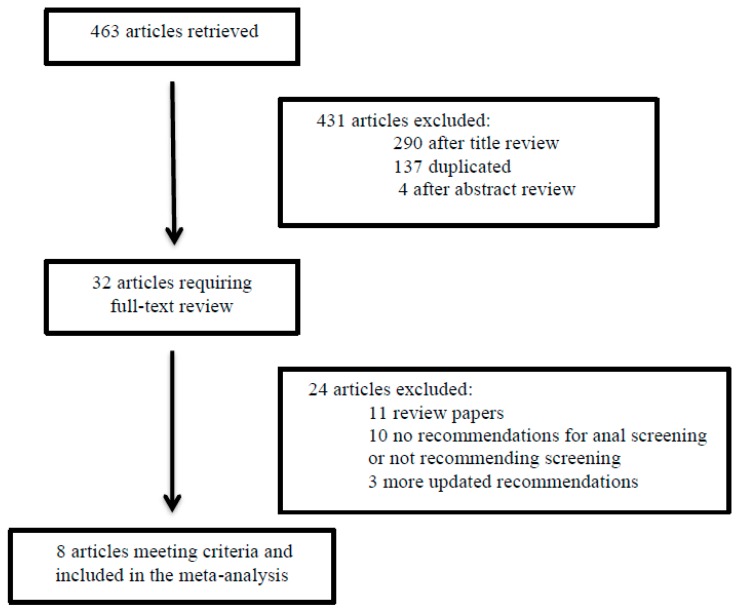
Flowchart study selection.

In total, 463 articles were retrieved in the database search. Of these, 431 were excluded and 32 required review of the full paper. For the final analysis, eight papers fulfilled the inclusion criteria and were selected (Figure 1). Five are from the United States of America (USA) [17,18,19,20,21] and three are from Europe [22,23,24]. Considering the European recommendations, one was from the European AIDS Clinical Society [22] and one each from Spain [23] and Germany/Austria [24]. Seven of the eight papers targeted HIV-positive patients [17,18,19,20,22,23,24], while the eighth focused exclusively on solid-organ transplant recipients [21]. In the case of HIV-positive patient recommendations [17,18,19,20,22,23,24], all of them included HIV-positive MSM as a screening group.

In 2007, the New York State Department of Health AIDS Institute [17] became the first to issue recommendations for yearly anal cytology in HIV-positive patients who are (1) MSM, (2) patients with a history of anogenital condylomas, and/or (3) women with abnormal cervical and/or vulvar histology (guidelines being updated). This was the only recommendation that specifically mentioned that HIV-positive patients should be screened regardless of their age. No mention was made in any of the other recommendations regarding upper and lower patient age limits. The same high-risk HIV-positive groups were also identified by the HIV Medicine Association of the Infectious Diseases Society of America [19] and by the Spanish AIDS Study Group [23]: MSM, history of anogenital warts, and a history of genital neoplasia (although in this case with some differences). The HIV Medicine Association of the Infectious Diseases Society of America [19] was the only one to specifically identify HIV-positive women with a history of receptive anal sex as a screening population.

The Northwest Pennsylvania Rural AIDS Alliance [18] and the German–Austrian guidelines [24] were the only two recommendations favoring screening of all HIV-positive patients.

Five of the recommendations for HIV-positive patients included considerations about cytology follow-up [17,18,22,23,24]. All favored at least annual cytology in case of a normal result. All five also suggested that an abnormal cytology result should be followed by anoscopy/HRA, with atypical squamous cells of undetermined significance (ASC-US) being the threshold in three recommendations [17,18,23], HSIL in one [24], and no definition of abnormality being given in one [22].

The only recommendation for screening populations other than HIV-positive patients was one for solid-organ transplant recipients, issued by the American Society of Transplantation Infectious Diseases Community of Practice [21]. This recommendation favored annual screening for transplant patients, targeting those with a history of receptive anal intercourse or cervical dysplasia. They further recommended that an abnormal anal cytology on screening (ASC-US as a threshold) should be followed by HRA.

Only the German–Austrian guidelines [24] included HPV testing as part of their follow-up management. Patients with high-risk HPV infection for more than one year were considered high-risk for anal cancer, and anoscopy (preferentially HRA) was recommended.

## 4. Discussion

The current incidence rate of anal SCC in HIV-positive MSM is higher than that of cervical cancer before the introduction of cervical cancer screening [25]. In many countries, routine screening has been implemented for cervical cancer, but not for anal screening in high-risk populations. Anal cancer screening was developed based on the cervical cancer screening process, given their similarities, as both exhibit HPV-related carcinogenesis. Several studies have shown that the sensitivity of anal cytology is similar to that of cervical cytology [13,14], although with a lower specificity [26]. A recent systematic review and meta-analysis described the performance of anal cytology (any abnormality as a threshold) to detect HSIL, in HIV-positive, with a sensitivity of 82% (95% Confidence Interval (CI), 74–87%) and specificity of 45% (95% CI, 44–66%), with a total of 18 studies included [27]. In a study by Albuquerque et al. [14], including 636 anal cytology samples and 323 biopsies, the sensitivity of anal cytology (any abnormality as the threshold) for predicting histological HSIL/cancer was 92% (95% CI, 78–97%) and the specificity 60% (95% CI, 48–71%), in immunosuppressed women with a history of anogenital tract neoplasia. 

Anal cytology is routinely performed as a liquid-based cytology, using a Dacron swab and, in contrast with the procedure for the cervix, collected blindly [28]. The recommended unsatisfactory sample rate in high-risk groups (e.g., HIV-positive MSM) should be <5% [29]. The Bethesda terminology is normally used for classification, as it is for the cervix [28]. A proper digital anorectal examination should always be performed to detect masses suggesting anal cancer that may be missed by cytology or by HRA [17]. 

Our study found that there are no national recommendations favoring anal cancer screening, but there are eight societies, institutes, or groups recommending screening using anal cytology. A major reason for the lack of national recommendations is the absence of randomized controlled trials showing that anal cancer screening prevents cancer appearance (e.g., by detecting and treating anal HSIL) and/or detection of cancers in an early stage. The Anal Cancer HSIL Outcomes Research (ANCHOR) study is currently ongoing in the USA. This is a randomized phase III trial comparing treatment of anal HSIL with active monitoring to prevent anal cancer in HIV-positive patients [30]. 

There are no specific recommendations for HIV-negative MSM or HIV-negative women with a previous history of genital neoplasia, and there is a single recommendation for screening solid-organ transplant recipients. HIV-negative MSM and solid-organ transplant recipients are also at an increased risk of anal SCC [4,8], although lower than that of HIV-positive patients. Several studies have provided evidence of associations between genital neoplasia, AIN, and anal SCC [31,32,33,34,35,36]. Women with a history of in situ or invasive gynecological neoplasm have a 13-fold increase in anal SCC [32]. Increases in the number and locations of affected genital sites were associated with a higher risk of anal precancerous lesions and seem to be higher in the vulva [36]. There are data consistently showing that these non-HIV groups have an increased risk of anal SCC, but there is a clear need for studies evaluating the benefit of anal cancer screening and its cost-effectiveness in these cohorts.

There were two recommendations for screening all HIV-positive patients [18,24]. Data on HIV-positive heterosexual men and HIV-positive women also indicate a higher incidence of anal SCC in those groups, although lower than that in HIV-positive MSM [37]. A study [37] involving 13 cohorts from North America and including 34,189 HIV-infected and 114,260 HIV-uninfected individuals showed that the unadjusted anal cancer incidence rates for HIV-positive MSM were 131 per 100,000 person-years; 46 per 100,000 person-years for HIV-positive heterosexual men and 30 per 100 000 person-years for HIV-positive women.

Three societies have specifically identified HIV-positive patients with anogenital condylomas as a risk group for screening [17,19,23]. Several studies have described that a significant percentage of anal condylomas in HIV-positive patients harbor high-grade lesions [38,39,40,41]. Individuals with condylomas also have a long-term increased risk of HPV-associated anogenital cancers [42].

Three of the included recommendations are from Europe [22,23,24], all for HIV-positive patients. There are other societies/agencies from European countries that have issued recommendations for anal cancer screening, but anal cytology was not recommended as a screening method. In France, a group of experts in HIV recommended screening in HIV-positive that are MSM, patients with history of condylomas, or women with a history of cervical lesions. This anal screening should include a proctological examination, digital anorectal examination, and anoscopy [43]. The Italian Society of Colorectal Surgery has recently published their practice parameters for the diagnosis and treatment of AIN. They stated that a clinical examination and HRA with biopsies of suspicious lesions are the most important tests for an appropriate diagnosis of AIN. The role of anal cytology, according to them, needs to be further clarified [44]. There are also other European associations that have guidelines for cancer prevention in HIV-positive patients, but that do not specifically recommendation anal cancer screening in this high-risk group, e.g., the Netherlands [45] and the United Kingdom (UK) [46].

Regarding the screening interval, in case of a normal result, the most common recommendation was to repeat cytology once a year. In case of an abnormal result, the recommendation (when available) was a referral to anoscopy/HRA. Having an ASC-US result as the threshold for referral can be important given the poor correlation between the cytology and histology grades [14,47] and the fact that anal cytology frequently underestimates histological results. A cost-effectiveness study by Goldie et al. [48] showed that anal cytology screening in HIV-infected MSM in all stages prolonged quality-adjusted life expectancy. For patients with a CD4 >0.5 X 10^9^/L, an anal cytology every two years had a better cost-effectiveness ratio, and for a CD4 <0.5 X 10^9^/L, yearly anal cytology was better due to a higher prevalence of disease [48]. This study from the USA [48] suggested that screening is cost-effective; however, studies [49,50] in the UK showed that anal cancer screening is unlikely to be cost-effective in HIV-positive MSM. This conflicting information is related to the paucity of data available for these analyses [51].

There were no indications of an appropriate lower age limit for screening. Only the New York State Department of Health AIDS Institute [17] addressed patient age; they suggested that screening should be offered regardless of age. There was also no indication in any of the published guidelines of an upper age limit for screening. In HIV-positive MSM, anal cancer incidence increases with age [52]. In a study by Colon-Lopez [52], if a 5-year cumulative incidence of anal cancer of 0.25% was considered the lower limit to target HIV-positive populations for screening, this threshold was only achieved for HIV-positive MSM and with specific age limits (for those with AIDS beginning at age ≥30 years and for those with HIV age ≥45 years).

HPV testing has not been routinely recommended as part of screening. In a meta-analysis [4], the prevalence of any anal HPV type was 92.6% in HIV-positive MSM. In this population, HPV testing has a high sensitivity but low specificity for anal HSIL due to this high prevalence [53].

Receptive anal sexual intercourse in men is a well-known risk factor for anal SCC [54], but in women, a consistent association has not been described [55]. The HIV Medicine Association of the Infectious Diseases Society of America has identified HIV-positive women with a history of receptive anal sex as a screening population [19]. A study by Gaisa et al. [56] has reported a high rate of anal HSIL in HIV-positive women who do not meet these guidelines (history of anal sex and genital neoplasia), suggesting that extending screening to all HIV-positive women might be more appropriate. 

Anal cytology does have important limitations, e.g., poor correlation with histology, limited sensitivity in some settings, and the possibility of false negative results [14]. High-resolution anoscopy is the “gold standard” for AIN detection but is expensive, invasive, and only available in limited settings, with a limited number of clinicians trained on it. Cytology is a less expensive method that is easier to perform by trained healthcare professionals, less invasive, and therefore potentially better suited as a screening method [14]. 

## 5. Conclusions

Our study has shown that, although there are no national recommendations favoring anal cancer screening, eight societies, institutes, or groups have described anal cytology as a possible screening method. These have focused on HIV-positive individuals, mostly MSM as a high-risk group. For HIV-positive patients, two recommendations called for screening in all patients, while the others only in specific HIV subgroups. Most of them included a follow-up plan according to the cytology result, but there was no indication as to when screening should be started or stopped. There is a need for trials evaluating the role of anal cancer screening in anal cancer prevention and/or early stage detection.

## Figures and Tables

**Table 1:** Recommendations for anal cytology as a method for anal cancer screening.

Society/Institute/Group	Year	Country	Target Population	Recommendation	Management after Cytology	Age	Grade	HPV Testing
New York State Department of Health AIDS Institute [17]	2007	USA	HIV-positive patients	Anal cytology at baseline in HIV-infected populations: - MSM - History of anogenital condylomas - Women with abnormal cervical and/or vulvar histology	- Normal cytology to be repeated annually. - Refer patients with abnormal results ≥ ASC-US for HRA.	Any age.	ND	ND
Northwest Pennsylvania Rural AIDS Alliance [18]	2011	USA	HIV-positive patients	Anal cytology at baseline in all HIV-positive.	- Normal cytology to be repeated annually, especially for HIV-positive MSM. - Patients with a low CD4+ T-cell count (<500 cells/mm3) should be monitored 6–9 months (author suggestion). - Refer patients with abnormal results ≥ ASC-US for HRA.	ND	ND	High and low-risk HPV. Not included in the algorithm management.
HIV Medicine Association of the Infectious Diseases Society of America [19]	2014	USA	HIV-positive patients	Anal cytology in HIV-positive patients: - MSM - Women with receptive anal sex - Women with abnormal cervical cytology - History of genital warts	ND	ND	Weak. Moderate quality evidence.	ND
The American Society of Colon and Rectal Surgeons [20]	2018	USA	High-risk populations: - HIV-positive - MSM - History of cervical dysplasia	Anal cytology may be considered in high-risk populations. Not for universal screening.	ND	ND	Weak. Moderate quality evidence, 2B.	HPV testing may be used as an adjunct to screening.
American Society of Transplantation Infectious Diseases Community of Practice [21]	2019	USA	Solid-organ transplanted patients	Anal cytology for solid-organ transplant patients: - History of receptive anal intercourse - History of cervical dysplasia	- Normal cytology to be repeated every 1–3 years. - Refer patients with abnormal results ≥ ASC-US for HRA.	ND	Weak. Low quality evidence.	ND
European AIDS Clinical Society [22]	2018	-	HIV-positive patients	Digital rectal exam ± anal cytology in HIV-positive patients: - MSM - Persons with anogenital HPV-associated dysplasia	- Normal cytology to be repeated in 1–3 years. - Patients with abnormal results should be referred for anoscopy.	ND	ND	ND
Spanish AIDS Study Group/Grupo de Estudio de SIDA (GeSIDA) [23]	2014	Spain	HIV-positive patients	Anal cytology in HIV-positive patients: - MSM - Women with cervical cancer or HSIL - History of anogenital condylomas	- Normal cytology to be repeated annually. - Refer patients with abnormal results ≥ ASC-US for HRA.	ND	ND	HPV PCR increases sensitivity. Not included in the algorithm management.
Several German Societies. Lead Management: Deutsche AIDS-Gesellschaft (DAIG)/German AIDS Society [24]	2015	Germany Austria	HIV-positive patients	Anal cytology in all HIV-positive patients	- Normal cytology to be repeated annually. -Refer patients with cytology HSIL for anoscopy (within 3 months). - Other cytology results to repeat cytology in 3–6 months. If the second is abnormal, to refer for anoscopy.	ND	ND	If necessary HPV typing. In case of high-risk HPV > 1 year, these are high-risk patients and should be submitted to anoscopy.

ASC-US: Atypical squamous cells of undetermined significance; HPV: Human papillomavirus virus; HRA: High-resolution anoscopy; HSIL: High-grade squamous intraepithelial lesions; MSM: Men who have sex with men; ND: Not described; PCR: polymerase chain reaction; USA: United States of America.
